# Spatial patterns of pulmonary tuberculosis (TB) cases in Zimbabwe from 2015 to 2018

**DOI:** 10.1371/journal.pone.0249523

**Published:** 2021-04-08

**Authors:** Isaiah Gwitira, Norbert Karumazondo, Munyaradzi Davis Shekede, Charles Sandy, Nicolas Siziba, Joconiah Chirenda

**Affiliations:** 1 Department of Geography Geospatial Sciences and Earth Observation, Faculty of Science, University of Zimbabwe, Harare, Zimbabwe; 2 National TB Control Program, Ministry of Health and Child Care, Harare, Zimbabwe; 3 Department of Community Medicine, Faculty of Medicine and Health Sciences, Parirenyatwa Hospital, University of Zimbabwe, Harare, Zimbabwe; The University of Tennessee Knoxville, UNITED STATES

## Abstract

**Introduction:**

Accurate mapping of spatial heterogeneity in tuberculosis (TB) cases is critical for achieving high impact control as well as guide resource allocation in most developing countries. The main aim of this study was to explore the spatial patterns of TB occurrence at district level in Zimbabwe from 2015 to 2018 using GIS and spatial statistics as a preamble to identifying areas with elevated risk for prioritisation of control and intervention measures.

**Methods:**

In this study Getis-Ord G_i_* statistics together with SaTscan were used to characterise TB hotspots and clusters in Zimbabwe at district level from 2015 to 2018. GIS software was used to map and visualise the results of cluster analysis.

**Results:**

Results show that TB occurrence exhibits spatial heterogeneity across the country. The TB hotspots were detected in the central, western and southern part of the country. These areas are characterised by artisanal mining activities as well as high poverty levels.

**Conclusions and recommendations:**

Results of this study are useful to guide TB control programs and design effective strategies which are important in achieving the United Nations Sustainable Development goals (UNSDGs).

## Background

Most disease-related deaths recorded in developing countries are associated with commonly occurring communicable diseases such as TB and Human Immunodeficiency Virus (HIV) [1, 2]. The WHO African region contributed 24% of the estimated 10 million *Mycobacterium tuberculosis* (*M*.*tuberculosis*) infections recorded globally in 2018 [3]. TB is an infectious bacterial airborne disease caused by *M*. *tuberculosis* complex and ranks among the top 10 causes of deaths in the world [3–5]. The disease is transmitted from person to person through breathing air contaminated by the bacteria [1]. TB is ranked above HIV/AIDs as the leading cause of death from a single infectious agent [6]. Most countries in sub-Saharan Africa (SSA) are characterised by high TB incidence rates of up to 275 cases per 100,000 of the population [7]. In an effort to reduce TB incidence, Zimbabwe adopted the global World Health Organization (WHO) ‘*End TB Strategy*’ which aims to reduce annual TB-related deaths by 95% and TB incidence by 90% at the end of 2035 compared to 2015 [3, 8]. This strategy is in line with United Nations Sustainable Development Goals (UNSDGs) particularly SDG 3 which targets to end the global TB epidemic by 2030 [9].

The success of national TB control programmes (NTP) in most parts of the developing world particularly in Africa is hindered by failure to consider spatial heterogeneity in the distribution of the disease [10]. Without evidence of fine scale spatial heterogeneity in the occurrence of the disease [11], uniform interventions are implemented across different settings by NTP. Thus, a better understanding of the spatial epidemiology of TB may guide policy makers in formulating effective prevention and control strategies [12–14]. For instance, the identification of TB clusters and hotspots provides the basis for targeted control of the disease as well optimizing resource allocation [10]. This is particularly important in resource limited countries such as Zimbabwe where there is need to prioritise allocation of resources by focusing on areas with the highest disease burden as they have the greatest need. Therefore, this study hypothesises that effective management of communicable diseases such as TB primarily depends on accurate detection and mapping of spatial heterogeneity in the occurrence of the disease. Achieving TB targets and goals at national or global scales require an understanding of the spatial pattern of the disease which acts as a preamble to developing effective strategies aimed at reducing new infections.

In the context of Zimbabwe, most research on TB has been largely limited to trend analysis of prevalence of the disease. For example, studies have focused on the prevalence of TB in Zimbabwe resulting from the economic crisis of 2008 [15] including the prevalence of drug resistant TB using non-spatial statistical analysis [16]. Other studies have focused on developing strategies to control TB such as the use of directly observed treatment (DOT) [17–19] while others assessed factors influencing adherence to TB treatment [20]. Although these studies have provided valuable insights in understanding risk factors of TB occurrence, there is limited application of spatial analysis particularly in detecting hotspots and cold spots of TB especially in Zimbabwe. This is despite the fact that GIS-based spatial analysis combined with spatial statistics are indispensable tools for supporting surveillance and control of most diseases in the world [21–23].

To date, GIS and spatial statistics have been applied to understand patterns of TB occurrence in several countries with different environmental settings [6, 7, 13, 22, 24, 25]. These studies have generated important information about the distribution of the disease and its transmission patterns. However, the major limitation of these studies is that they are biased towards smaller spatial scales such as a single urban area over short temporal durations [14, 26]. By focussing on smaller spatial scales, these studies fail to capture the spatial pattern of TB at a spatial scale that is relevant for TB programming, for example district scale. The district is the spatial epidemiological administrative unit at which TB interventions and control are planned in Zimbabwe [27]. In this regard, spatial analysis becomes relevant when the spatial unit at which TB data is analysed represents the expected epidemiological dynamics in the country. Thus, it is important to fully understand the spatial heterogeneity in the occurrence of TB at large spatial scales such as the district level to inform targeted public health response, which is regarded as one of the most effective approaches in disease control [28].

Although GIS and spatial statistics have been successfully applied to identify TB hotspots and clusters, most of these studies have applied either of these techniques in isolation despite their complementarity as well as their potential to reduce uncertainties associated with adoption of a single method to guide control interventions [29]. This limitation calls for the adoption of multiple cluster detection methods as this is critical in the detection of truly representative high-risk areas. The application of multiple techniques is increasingly recommended in spatial epidemiological studies to eliminate the limitations of a single method in disease hotspot detection [30–32]. This study therefore aimed at characterising the spatial pattern of TB hotspots and clusters using geospatial techniques and spatial statistical tools based on TB notification data recorded at district level from 2015 to 2018 in Zimbabwe.

## Methods

### Study area

**Fig 1:**
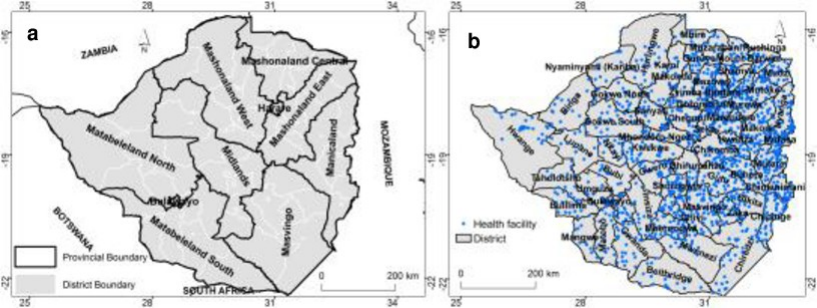
a) Location of the study area including the administrative Provinces and districts and b) Spatial distribution of health facilities where TB cases were recorded.

The study was conducted in Zimbabwe located in southern Africa between latitudes 15.5˚ and 22.5˚ S and longitudes 25˚ and 33˚E [33]. Zimbabwe is characterized by a subtropical climate with distinct seasons [34]. The country is divided into ten administrative provinces and sixty-six (66) districts (Fig 1A). The total human population was estimated at 14.64 million in 2019 *https*:*//data*.*worldbank*.*org* [35]. Of the 14.64 million, 67% of the population resides in rural areas. The greater proportion of the population is at risk from the three top killer diseases i.e., HIV, TB and malaria. A network of health infrastructure has been constructed to provide health services across the country (Fig 1B)

### Data sources

#### TB notification data

Aggregated data on TB notification from 2015 to 2018 were obtained from the Ministry of Health and Child Care (MOHCC) head office in Zimbabwe. The country has a well-established WHO-recommended directly observed treatment, short-course (DOTS)-based national TB programme (NTP) in which TB treatment services are integrated within the general primary health care delivery system [36]. The health care system includes rural hospitals, district, provincial and mission hospitals (Fig 1B). TB is a notifiable disease in Zimbabwe hence a person with TB is required by law to report to the nearest health centre for treatment due to the infectiousness of the disease. The assumption in this study was that every health facility is manned by well trained health personnel who can accurately test for TB. The data used in this study are aggregated district totals from the rural health centres (RHC/clinics) and thus did not include the physical address of the patients [37]. The diagnosis of TB in Zimbabwe follows national guidelines and is mostly based on chronic cough, or suggestive clinical symptoms (fever, night sweats, weight loss), and positive sputum smears and/or chest X-Ray [15] including bacteriological confirmation using Gene Xpert [38]. After TB diagnosis is completed at each health facility, heath workers complete notification forms and patient treatment cards for all recurrent patients. The notification information includes diagnosis, patient category, treatment regimen and monitoring indices [39]. This information is then entered into the DOTS register at each facility and later uploaded into the District Health Information Management System (DHIS 2) [38]. In this study, a patient with TB relapse was considered to belong to any of the following categories: treatment after failure, treatment after default or retreatment based on the WHO TB case definition [40]. A relapse may also be a patient who completed treatment but shows smear-positive symptoms while patients treated after failure represent those who failed first line treatment. In addition, patients who default from treatment and present again with smear-positive pulmonary TB (PTB) are classified as treatment after default while retreatment other refers to all other recurrent TB cases [40]. Since the study used anonymized data abstracted from district registers there was no need for ethics approval or informed patient consent. However, permission to use the routinely collected data was obtained from the NTP under the Ministry of Health and Child Care.

### Population data

Population data used in this study were obtained from the Zimbabwe Statistics Agency (ZIMSTAT) based on the 2012 population census as well as from projections for the years 2015 and 2018. The 2015–2018 population figures were obtained using a medium scenario based on the projected annual growth rate of 1.1% [41]. Population projection data were used for the years 2015–2018 because in Zimbabwe, a national population census is conducted after every 10 years hence the population of intercensal years is estimated based on the projected growth rate. The population projections were carried out assuming geometric growth in the population [41].

### Data analysis

#### Global autocorrelation of TB cases

The aggregated TB notification data at district level were used to calculate TB incidence by dividing the total TB notification for each district for a particular year by the projected population of that given year before multiplying by 100, 000. This gives a TB incidence per 100,000 of the population. The level of spatial clustering of TB cases at district level was examined by applying spatial autocorrelation using global Moran’s *I* [42] statistic with row standardized inverse distance weight matrices. The technique tests whether there is any systematic pattern in the distribution of TB notifications among districts of Zimbabwe as opposed to being randomly distributed. The index ranges from -1 to +1 with a score of zero indicating no clustering while a positive value suggests that the distribution of TB cases in neighbouring districts is more spatially aggregated than a random pattern [43, 44]. In contrast, a negative value suggests dispersion of TB cases at the district level. Dispersion of TB cases means that districts with similar TB cases are actually less likely to be located near each other [45]. The TB cases and the projected population were spatially joined to the geometry of districts in a GIS for mapping using the spatial join function in Arcmap 10.3 [46].

#### Local spatial clusters of TB cases

Global techniques of spatial autocorrelation such as the Moran’s *I* can successfully detect spatial clustering, but they fail to specify the location of the clusters [47]. Therefore Getis-Ord *G*_i_^*^ was used to examine where TB cases formed statistically significant local aggregation in geographic space [48, 49]. The scan statistic method by Kulldorff (SaTScan 9.6) [50] was used to further detect the location as well as extent of TB clustering across districts of Zimbabwe. The following sections outline how each of these methods were implemented to detect clustering of TB cases at district level.

#### Detecting local spatial clusters using Getis-Ord G_i_^*^

The Getis-Ord G_i_* was used to obtain additional information on the spatial pattern of TB such as the intensity and stability of core hotspot or coldspot areas [48]. The Getis-Ord Gi* method considers each district in the context of neighbouring districts which suggests likelihood of spatial autocorrelation among neighbouring districts [48, 49]. The conceptualization of spatial relationships was based on contiguity edges corners (*also referred to as the Queen’s case*) available in ArcGIS 10.1.3 software where districts that share an edge or corner were considered as neighbours. The *Queen’s* case was adopted to analyse spatial adjacency relationships since it compensates for the irregular size and shape of the districts [51] and is most ideal when modelling contagious diseases such as TB [46]. Based on the neighbours of a district, the ratio of the local sum of TB cases within neigbouring districts was compared to the sum of TB cases in the whole study area. Where the local sum of TB cases was significantly different from the expected local sum, and that difference was too large to be the result of random chance, the result was regarded as statistically significant based on the Z-score [52].

Since the Getis-Ord G_i_* considers neighbouring districts, the results are likely to be correlated hence the need to account for multiple testing and spatial dependency to accurately detect the pattern of TB [53]. Spatial dependence arises from the geometric relationship among districts that share a common boundary in addition to the TB cases recorded in neigbouring districts. Although the Bonferron method is most widely used to account for multiple testing and spatial dependency, it has a limitation of being conservative [53]. To overcome this limitation, the false discovery rate (FDR) method available in ArcGIS 10.3.1 was applied in this study. The FDR correction accounts for multiple testing and spatial dependency by controlling for the average rate that identified hotspots are truly significant [54]. This method has been shown to accurately evaluate occurrence of hotspots [55].

The detected significant hotspots/coldspots imply high/low TB cases in a particular district are surrounded by districts with high/low TB cases, respectively [48, 56]. To classify a district as a hotspot, a threshold Z-score of > 1.96 and a P-value of < 0.05 were considered [12]. Districts with Z-scores between -1.96 and +1.96 were considered as having non-significant clusters while those with Z-scores <-196 were classified as coldspots.

The Getis-Ord G_i_* takes the form;

Gi*(d)=∑jwij(d)Xj−wi*X¯*S*{[(nS1i*)−wi*2]/(n−1)}12 (1)

where:

X_j_ = TB Case for district *j*

W_ij_ = spatial weights between district *i* and *j*

n = total number of districts

W_ij_ (d) = spatial weights vector with values for all districts j within distance d of district i

W_*i*_ * = sum of weights

S1_i_* = sum of squared weights

s* = standard deviation of data cells

#### Detecting local spatial clusters using SaTscan

The scan statistic method developed by Kulldorff (SaTScan 9.6) [50] was applied to detect the spatial pattern of TB at the district level. The method has been widely used for detecting spatial clusters of diseases in different environmental settings [57–59]. Purely spatial analysis based on the discrete poisson method was applied to detect clusters of varying sizes at different locations including their relative risk (RR) and a significance value generated using Monte Carlo Simulations [60]. The relative risk for each cluster was calculated by comparing the observed and expected TB cases in each circular window resulting in a log likelihood ratio [61]. In this study, the number of TB cases aggregated at district level, the projected population and the centroid coordinates of each district were used as input files. The discrete Poisson model which assumes that the number of TB cases in each district followed a Poisson distribution with a known population at risk was applied to detect significant TB clusters. The population at risk for each of the four years was derived from population projections based on the 2012 census. The Gini coefficient [62] was used to determine the maximum reported cluster size as a percentage of the population for the respective years (Table 1). For the four years, the maximum reported cluster size ranged from 6–20% of the population at risk.

**Table 1:** Maximum reported cluster size (MRCS) from 2015–2018 in Zimbabwe determined using the Gini coefficient.

Year	% of the Population	Optimal Gini Coefficient
2015	6	0.2784
2016	10	0.1956
2017	10	0.1949
2018	20	0.2179

The Gini coefficient is an intuitive way to evaluate the degree of heterogeneity among a collection of clusters [62].

## Results

**Fig 2:**
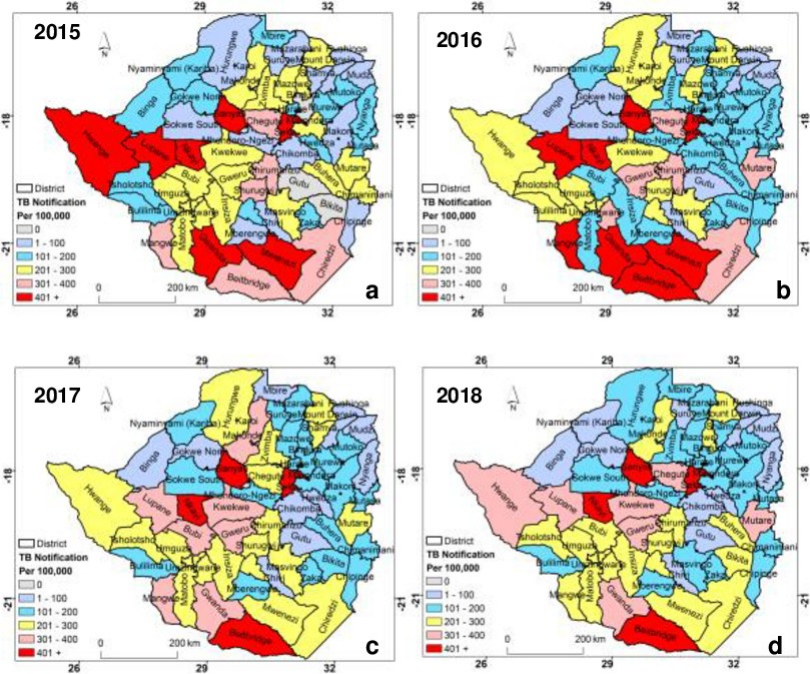
Spatial distribution of TB notification per 100,000 of the population at district level in Zimbabwe for (a) 2015, (b) 2016, (c) 2017 and (d) 2018.

The spatial distribution of TB notification rates in Zimbabwe at district level from 2015 to 2018 is illustrated in Fig 2. The TB notification is relatively higher in the central and southern part of the country than in the east and north western regions. In fact, across all the years considered in this study, TB notification was consistently high in the central and southern regions. The TB notification was high in Beitbridge, Buhera, Chirumhanzu, Gwanda, Hwange, Mwenezi and Sanyati districts (Fig 2).

The results of Moran’s *I* analysis showed a strong positive spatial autocorrelation (P< 0.05) across all the four years from 2015 to 2018. Specifically, results show that the Moran’s index was 0.173, 0.140, 0.243 and 0.032 for 2015, 2016, 2017 and 2018 respectively. The significant positive spatial autocorrelation indicates that the distribution of TB cases was more spatially clustered than would be expected in a random process for each year across the districts of Zimbabwe.

### Results of hotspot analysis

**Fig 3:**
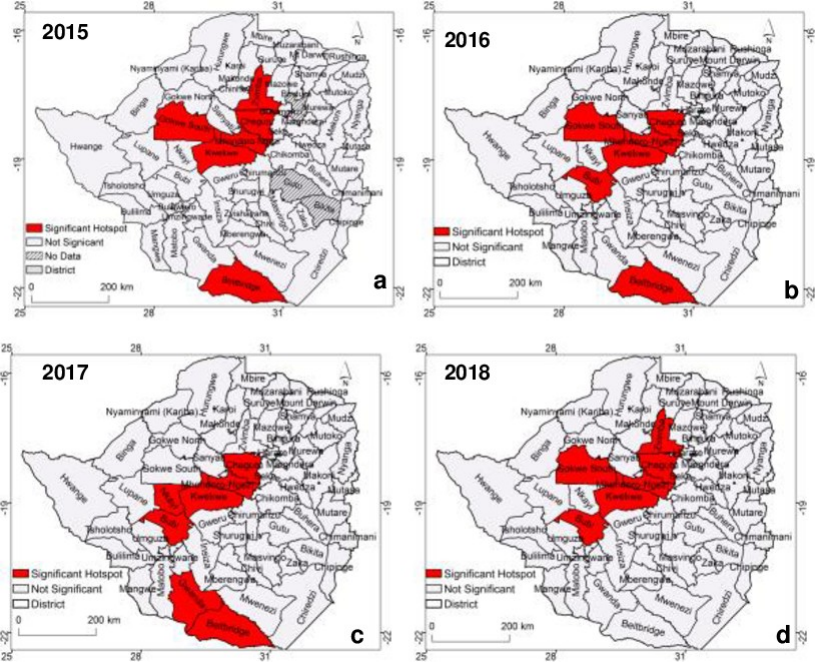
Spatial distribution of TB hotspots in Zimbabwe at district level for (a) 2015, (b) 2016, (c) 2017 and (d) 2018.

Results of hotspots analyses using local Getis Ord G_i_* after applying the false discovery rate are illustrated in Fig 3. The results show that significant TB hotspots (Z-score>1.96) are characteristic of the central, southern and western part of the country. These hotspots covered most urban areas and districts characterised by both formal and informal mining activities such as Kwekwe, Mhondoro-Ngezi and Chegutu (Fig 3). Over the study period, Chegutu, Kwekwe and Mhondoro-Ngezi and were persistently characterised by significant hotspots (Fig 3). Most of these districts in the centre of the country lie along the Great Dyke of Zimbabwe where small scale artisanal miners predominate. While hotspots of TB were observed in the central and southern part of the country the majority of the county was characterised by non-significant TB hotspots (Z-score<1.96) across the four years (Fig 3).

### Results of cluster detection using SaTscan

**Fig 4:**
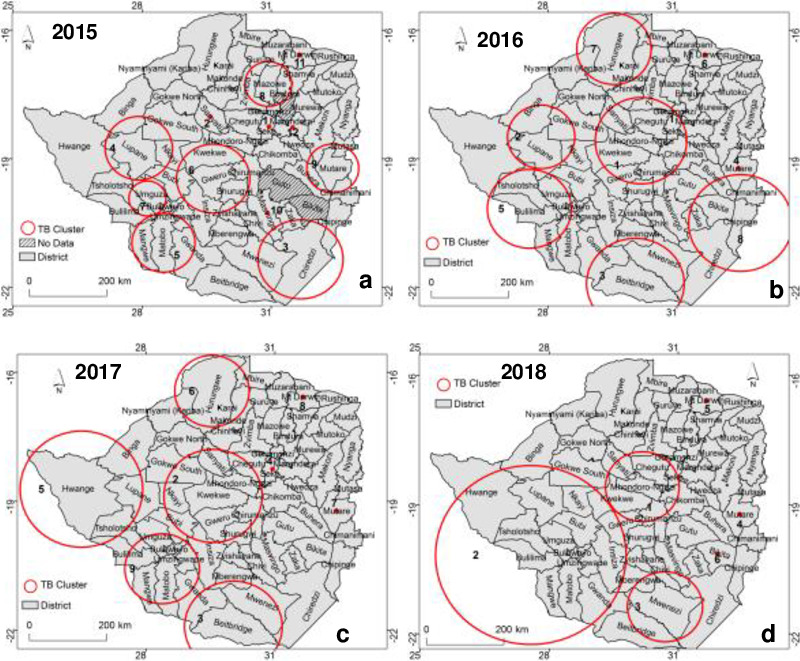
Spatial distribution of statistically significant primary and secondary TB clusters detected by purely spatial analysis based on the Gini coefficient for a) 2015, b) 2016, c) 2017 and d) 2018.

The TB epidemic in Zimbabwe is characterised by geographically distinct clusters across the study area (Fig 4). Using spatial analysis, different clusters were identified for each year with the year 2015 having the highest number of clusters centred on specific districts (Fig 4). In general, the pattern of TB occurrence exhibits stability or persistence for some regions e.g., central and southern regions which are characterised by significant clusters in each year considered in this study. Overall, 25 significant clusters (P < 0.001) were detected and their characteristics are summarised in Table 2.

**Table 2:** Significant spatial clusters of TB in Zimbabwe based on maximum reported cluster size from 2015–2018.

Year	Cluster #	District	Radius	# of Locations	LLR	Pvalue	Observed	Expected	RR
	1	Seke	0	1	904.05	0.001	1075	210	5.29
	2	Sanyati	0	1	771.97	0.001	1024	223	4.74
	3	Chiredzi	102	2	687.02	0.001	2295	970	2.50
	4	Lupane	82	2	269.10	0.001	968	418	2.37
	5	Matobo	76	3	175.84	0.001	1135	621	1.87
**2015**	6	Gweru	89	4	159.26	0.001	2147	1443	1.53
	7	Umguza	45	2	131.78	0.001	2137	1491	1.47
	8	Mazowe	58	3	67.12	0.001	1766	1335	1.35
	9	Mutare	63	2	40.85	0.001	1165	888	1.33
	10	Masvingo	0	1	17.57	0.001	766	615	1.25
	11	Mt Darwin	0	1	3.76	0.02	494	436	1.14
	12	Marondera	0	1	2	0.01	410	371	1.10
	1	Mhondoro-Ngezi	112	8	620.61	0.001	4434	2579	1.86
	2	Lupane	82	2	405.73	0.001	1143	435	2.70
	3	Beitbridge	117	3	380.88	0.001	1822	899	2.10
**2016**	4	Mutare	0	1	132.36	0.001	767	403	1.93
	5	Bulima	102	5	100.36	0.001	2800	2144	1.34
	6	Mt Darwin	0	1	15.37	0.001	580	458	1.27
	7	Hurungwe	92	2	13.75	0.001	1207	1037	1.17
	8	Chipinge	125	5	6.62	0.001	2496	2326	1.08
	2	Kwekwe	120	9	714.10	0.001	4561	2569	1.94
	3	Beitbridge	117	3	305.04	0.001	1649	852	2.00
	4	Seke	0	1	267.41	0.001	626	212	3.00
**2017**	5	Hwange	150	3	70.46	0.001	931	618	1.53
	6	Hurungwe	92	2	50.08	0.001	1316	992	1.34
	7	Mutare	0	1	34.13	0.001	554	383	1.46
	8	Mt Darwin	0	1	33.94	0.001	617	436	1.43
	9	Insiza	90	5	24.28	0.001	2546	2225	1.16
	1	Mhondoro-Ngezi	90	6	1254.75	0.001	4415	1953	2.49
	2	Bulilima	231	14	665.80	0.001	6961	4559	1.69
**2018**	3	Mwenezi	90	2	127.49	0.001	1099	656	1.70
	4	Mutare	0	1	111.54	0.001	774	432	1.81
	5	Mt Darwin	0	1	24.45	0.001	653	492	1.34
	6	Bikita	0	1	18.12	0.001	494	373	1.33

RR: Relative risk, LLR: likelihood ratio.

The summary of TB clusters detected using the Gini coefficient and their characteristics illustrate that the most likely cluster with the highest relative risk was detected in 2015 while the lowest relative risk was recorded in 2018 (Table 2).

## Discussion

Results of this study indicated that TB exhibits spatial heterogeneity as indicated by the occurrence of spatial clusters as well as hotspots of the disease in specific districts. The identification of clusters and hotspots in similar locations by the two approaches adopted here i.e., SaTscan and Getis Ord G_i_*statistic suggest that they are viable and robust options for identifying and detecting areas of unusually high TB occurrence [12]. This demonstrates the advantages of utilising more than one spatial technique to understand spatial variability in disease occurrence which increases confidence in the validity and application of the results [43]. In studies where Getis Ord G_i_*statistic and SaTscan have been applied, the results have been used to guide TB interventions [24]. The use of GIS and spatial statistics in spatial epidemiological is an important approach for investigating infectious diseases and has been used to study diseases such malaria [60], breast cancer [57] and foot-mouth disease [61]. In these studies, the two techniques yielded useful information for guiding disease control.

The results of this study indicate that statistically significant hotspots and spatial clusters were common in central and southern parts of Zimbabwe. These regions are dominated by large urban areas such as Bulawayo, Gweru and Kwekwe and other rural districts characterised by formal and illegal mining (predominantly artisanal) activities. The occurrence of TB hotspots and clusters in these areas may be associated with high population resulting in overcrowding hence ease transmission of the disease. These results are similar to findings by Chirenda [14] who found that TB hotspots and clusters are common in urban areas due to large populations and inadequate health care services. Consistently across the four years, and especially in 2017, the districts along the Great Dyke exhibited TB hotspots. The Great Dyke region is rich in minerals and has a high concentration of illegal and commercial mining activities. As illustrated by previous studies [26, 63], mining activities expose miners to silica dust which increases the vulnerability of the populations to TB. Characteristics that increase the risk of small scale artisanal miners are reduced access to health care services, inadequate personal protective equipment and the relatively young age. Other health challenges like sexually transmitted infections and HIV infection have been found to be common among this population. All these further confound the increased risk of hotspot and clustering observed in this study.

The tendency of TB cases to cluster in specific localities is of interest to public health response. Significant interventions such as targeted active case finding, early treatment and DOTS could focus on these hotspots. As artisanal miners are a highly mobile population, interventions such as the Zimbabwe National TB Programme could prioritise its targeted active case finding using mobile clinics along the Great Dyke area. Although this study identified critical areas for TB interventions, it did not explore factors explaining the observed patterns. Thus, further research on the possible environmental or social determinants of spatial pattern of TB in Zimbabwe is strongly recommended.

An important result from this study is an observed decrease in relative risk associated with TB clusters over the four year period. The result implies that there has been a general decrease in number of TB notifications in Zimbabwe which suggests the effectiveness of interventions implemented in the general population to reduce TB transmission. The declining TB case notification rates also coincided with the scale-up of antiretroviral therapy in Zimbabwe [64]. The scaling up of antiretroviral therapy under the UNAIDS 90-90-90 targets is likely to result in continued decline in TB case notification rates [39, 64].

This study showed that southern districts such as Chiredzi and Beitbridge had high clustering but relatively low hotspot occurrence compared to its neighbours. Several plausible explanations could be proffered to explain this observation. First, Beitbridge is a border town characterised by high population movements between South Africa and Zimbabwe and is considered the gateway to South Africa. High migration activities might explain the volume of cases reported in Beitbridge district but may not necessarily explain transmission. Secondly, Chiredzi and Mwenezi districts which consistently showed high TB clusters throughout the four years and these are sugarcane growing districts. Studies describing high TB prevalence in agricultural settings are few and the findings of this study require further interrogation to assess the contribution of the agricultural sector to the burden of TB. However, because of the high economic activities from the agricultural business, there could be high local transmission and immigration associated with overcrowding in these areas [65]. Although the potential risk factors that can explain the clustering of TB were not explored in this study, previous studies demonstrated that the TB epidemic in Zimbabwe is largely driven by HIV [64]. Interestingly, areas where TB clusters were detected coincide with areas where clusters of HIV prevalence were also detected [66] suggesting possible co-infection by these two diseases [39].

The use of spatial statistics to explore the distribution and aggregation of TB may be useful in understanding the pattern of TB hotspots [12]. Therefore, TB interventions may not be able to use the “*one size fits all*” approach across different districts. Districts with high disease rates as a result of the social determinants of disease will require more focussed attention than areas with a low risk [21]. Areas where hotspots and clusters of TB cases were detected may be used as priority areas for targeted control to achieve high impact [44, 29]. Thus, to achieve the set targets under the UNSDGs there is need to implement more effective and stronger measures to control TB transmission in these clusters. Policymakers and health authorities will need to strengthen resource mobilization for improved TB prevention and control measures.

The strengths of this study is that the detection of TB hotspots and clusters were performed at district level which is the spatial epidemiological unit at which interventions are planned hence they are likely to be more relevant to policymakers. Performing analysis at district level improves our understanding of the spatial pattern of TB at a wider geographic scale in contrast to most studies which mostly focussed on urban areas [67]. The four years considered in this study may also be sufficient to assess the spatial pattern and stability of hotspots and clusters of the disease in the country as previous studies have focussed on a short time period of about a year. The utility of SaTscan and Getis-Ord G_i_* in differentiating districts with high incidence of TB cases, from those districts where active transmission may actually be common is significant. This may indicate that focussing resources in districts showing high incidence of TB disease may not address transmission with the associated risk factors. We therefore recommend that the NTP complements the surveillance data with geospatial techniques to identify districts with active transmission from those with cold spots.

Although scan statistics and Getis were successfully used to understand TB clusters and hotspots, there are limitations associated with this study. The TB cases reported in each district may be affected by under reporting which influences the detection of hotspots and clusters [12]. Under reporting is a major limitation for all studies that use TB notification data particularly in low income countries without health information management systems [68]. TB notification is dependent on programmatic responses such as ability to test, diagnose and treat individuals including accessibility of heath care services. Some previous studies have found that high TB notification rates in some areas may be associated with better access to TB diagnostic services rather than increased burden or transmission [68]. This may be common in areas with sparse health services. Under reporting impacts negatively on the capacity to accurately evaluate the epidemiology of the disease [68]. However, TB notifications are an important source of information in the context of local health systems as they can be used as a basis for intervention or programmatic efforts. The information obtained from spatial analysis of TB notifications can be used to prioritise areas requiring further supervision and to tailor interventions to local needs.

A potential drawback in the detection of hotspots in this study is data timeliness. Most of the heath data takes time to compile making it difficult for relevant information to be extracted on time to guide interventions. However, through this retrospective study useful insights on the spatial dynamics of TB occurrence were generated across districts of Zimbabwe. Another potential limitation of this study is that the district TB notification rates are based on data recorded at each health facility. It is therefore possible that a patient can visit more than one health facility resulting in double counting which introduces bias and subsequently affects the detection of TB hotspots and clusters. This is likely to happen in instances where health facilities are in close proximity to each other as well as in referral cases. This means the data recorded in such areas are unlikely to be independent of each other which increase the risk of over-reporting.

Furthermore, TB clusters detected in this study were assumed to be circular and given the irregularity of the administrative boundaries of districts in Zimbabwe, this could result in the exclusion of districts with excess risk. Moreso the results of this study are population-based making it difficult to apply them at individual level. Despite these limitations, this study applied GIS and spatial statistics to identify TB hotspots and clusters at the district level which is important for decision making and resource allocation. Future research need to focus on relationship between TB occurrence and various socio-economic and environmental risk factors to enhance understanding of the pattern of the disease.

## Conclusion

GIS and scan statistics were successfully applied to determine the extent to which TB cases were clustered in space at district level in Zimbabwe. The observed hotspots and clustering in districts with intense small scale mining activities may indicate social determinants of TB disease aiding high transmission. The information generated from this study is useful in providing detailed knowledge on the spatial pattern of TB occurrence which is critical for targeted TB interventions. This is particularly important in developing countries where there is need to prioritise allocation of limited resources by focussing on areas with greatest need which results in high impact in terms of disease control.

## Supporting information

S1 Data (XLS)

S1 Annex Districts of Zimbabwe showing the projected population, recoded TB cases and the notification rate from 2015 to 2018. (DOCX)
