# Supplementary material for: Spatial patterns of pulmonary tuberculosis (TB) cases in Zimbabwe from 2015 to 2018
Source: PLoS One. 2021 Apr 8;16(4):e0249523. doi: 10.1371/journal.pone.0249523 (PMC8031317; doi:10.1371/journal.pone.0249523)
Supplement: S1 Annex — (DOCX) [file pone.0249523.s002.docx]

S1 Annex. Districts of Zimbabwe showing the projected population, recoded TB cases and the Notification rate from 2015 to 2018

| **#** | **District** | **X** | **Y** | **Year** | **Proj_Pop** | **Cases** | **notif_rate** | **Year** | **Proj_Pop** | **Cases** | **notif_rate** | **Year** | **Proj_Pop** | **Cases** | **notif_rate** | **Year** | **Proj_Pop** | **Cases** | **notif_rate** |
| --- | --- | --- | --- | --- | --- | --- | --- | --- | --- | --- | --- | --- | --- | --- | --- | --- | --- | --- | --- |
| 1 | Beitbridge | 30.04 | -21.91 | 2015 | 124401 | 410 | 330 | 2016 | 125023 | 503 | 402 | 2017 | 125648 | 699 | 556 | 2018 | 138213 | 581 | 420 |
| 2 | Bikita | 31.91 | -20.15 | 2015 | 167594 | 0 | 0 | 2016 | 169605 | 315 | 186 | 2017 | 171640 | 278 | 162 | 2018 | 188804 | 494 | 262 |
| 3 | Bindura | 31.31 | -17.24 | 2015 | 174739 | 499 | 286 | 2016 | 177011 | 250 | 141 | 2017 | 179312 | 320 | 178 | 2018 | 197243 | 405 | 205 |
| 4 | Binga | 27.66 | -17.79 | 2015 | 140155 | 146 | 104 | 2016 | 140856 | 91 | 65 | 2017 | 141560 | 60 | 42 | 2018 | 155716 | 115 | 74 |
| 5 | Bubi | 28.66 | -19.53 | 2015 | 63125 | 132 | 209 | 2016 | 63441 | 107 | 169 | 2017 | 63758 | 198 | 311 | 2018 | 70133.8 | 154 | 220 |
| 6 | Buhera | 31.88 | -19.45 | 2015 | 254685 | 595 | 234 | 2016 | 257469 | 435 | 169 | 2017 | 260301 | 421 | 162 | 2018 | 286331 | 582 | 203 |
| 7 | Bulawayo | 28.55 | -20.14 | 2015 | 665559 | 1900 | 285 | 2016 | 668887 | 1748 | 261 | 2017 | 672231 | 1664 | 248 | 2018 | 739454 | 2137 | 289 |
| 8 | Bulilima | 27.57 | -20.16 | 2015 | 92125 | 176 | 191 | 2016 | 92586 | 188 | 203 | 2017 | 93049 | 148 | 159 | 2018 | 102354 | 188 | 184 |
| 9 | Centenary | 31.14 | -16.43 | 2015 | 125497 | 124 | 99 | 2016 | 127128 | 145 | 114 | 2017 | 128781 | 193 | 150 | 2018 | 141659 | 214 | 151 |
| 10 | Chegutu | 30.40 | -18.14 | 2015 | 156755 | 474 | 302 | 2016 | 159420 | 489 | 307 | 2017 | 162130 | 487 | 300 | 2018 | 178343 | 619 | 347 |
| 11 | Chikomba | 31.10 | -18.89 | 2015 | 127447 | 18 | 14 | 2016 | 129614 | 221 | 171 | 2017 | 131817 | 93 | 71 | 2018 | 144999 | 129 | 89 |
| 12 | Chimanimani | 32.72 | -19.78 | 2015 | 138273 | 291 | 210 | 2016 | 139794 | 209 | 150 | 2017 | 141332 | 170 | 120 | 2018 | 155465 | 160 | 103 |
| 13 | Chipinge | 32.48 | -20.50 | 2015 | 310828 | 130 | 42 | 2016 | 314274 | 574 | 183 | 2017 | 317731 | 486 | 153 | 2018 | 349504 | 574 | 164 |
| 14 | Chiredzi | 31.68 | -21.34 | 2015 | 318636 | 1255 | 394 | 2016 | 322460 | 1040 | 323 | 2017 | 326330 | 862 | 264 | 2018 | 358963 | 807 | 225 |
| 15 | Chirumhanzu | 30.49 | -19.32 | 2015 | 83544 | 292 | 350 | 2016 | 84379 | 337 | 399 | 2017 | 85223 | 228 | 268 | 2018 | 93745 | 232 | 247 |
| 16 | Chitungwiza | 31.06 | -18.01 | 2015 | 356840 | 361 | 101 | 2016 | 392524 | 194 | 49 | 2017 | 431776 | 303 | 70 | 2018 | 474954 | 267 | 56 |
| 17 | Chivi | 30.58 | -20.50 | 2015 | 172334 | 97 | 56 | 2016 | 174402 | 161 | 92 | 2017 | 176495 | 143 | 81 | 2018 | 194145 | 160 | 82 |
| 18 | Gokwe North | 28.83 | -17.57 | 2015 | 252399 | 283 | 112 | 2016 | 254923 | 236 | 93 | 2017 | 257472 | 203 | 79 | 2018 | 283219 | 264 | 93 |
| 19 | Gokwe South | 28.64 | -18.21 | 2015 | 316560 | 229 | 72 | 2016 | 319726 | 297 | 93 | 2017 | 322923 | 337 | 104 | 2018 | 355215 | 457 | 129 |
| 20 | Goromonzi | 31.34 | -17.79 | 2015 | 235492 | 0 | 0 | 2016 | 239495 | 333 | 139 | 2017 | 243566 | 312 | 128 | 2018 | 267923 | 429 | 160 |
| 21 | Guruve | 30.57 | -16.33 | 2015 | 128345 | 109 | 85 | 2016 | 130013 | 99 | 76 | 2017 | 131703 | 137 | 104 | 2018 | 144873 | 128 | 88 |
| 22 | Gutu | 31.25 | -19.61 | 2015 | 210948 | 0 | 0 | 2016 | 213479 | 198 | 93 | 2017 | 216041 | 153 | 71 | 2018 | 237645 | 210 | 88 |
| 23 | Gwanda | 29.16 | -21.24 | 2015 | 138839 | 619 | 446 | 2016 | 139533 | 590 | 423 | 2017 | 140231 | 524 | 374 | 2018 | 154254 | 543 | 352 |
| 24 | Gweru | 29.65 | -19.46 | 2015 | 258978 | 723 | 279 | 2016 | 261568 | 920 | 352 | 2017 | 264184 | 990 | 375 | 2018 | 290602 | 1028 | 354 |
| 25 | Harare | 31.07 | -17.86 | 2015 | 2168204 | 3495 | 161 | 2016 | 2192054 | 3328 | 152 | 2017 | 2216167 | 3311 | 149 | 2018 | 2437784 | 3339 | 137 |
| 26 | Hurungwe | 29.54 | -16.44 | 2015 | 341512 | 68 | 20 | 2016 | 347318 | 733 | 211 | 2017 | 353222 | 784 | 222 | 2018 | 388544 | 444 | 114 |
| 27 | Hwange | 26.50 | -18.72 | 2015 | 101762 | 445 | 437 | 2016 | 102271 | 305 | 298 | 2017 | 102782 | 304 | 296 | 2018 | 113060 | 410 | 363 |
| 28 | Hwedza | 31.64 | -18.74 | 2015 | 70968 | 116 | 163 | 2016 | 78064.8 | 110 | 141 | 2017 | 85871.28 | 69 | 80 | 2018 | 94458 | 81 | 86 |
| 29 | Insiza | 29.41 | -20.26 | 2015 | 101297 | 207 | 204 | 2016 | 101803 | 172 | 169 | 2017 | 102312 | 209 | 204 | 2018 | 112543 | 296 | 263 |
| 30 | Kariba | 28.61 | -16.89 | 2015 | 71697 | 115 | 160 | 2016 | 72916 | 98 | 134 | 2017 | 74156 | 104 | 140 | 2018 | 81572 | 80 | 98 |
| 31 | Kwekwe | 29.56 | -18.87 | 2015 | 284644 | 822 | 289 | 2016 | 287490 | 779 | 271 | 2017 | 290365 | 897 | 309 | 2018 | 319402 | 1039 | 325 |
| 32 | Lupane | 27.92 | -18.86 | 2015 | 100354 | 407 | 406 | 2016 | 100856 | 507 | 503 | 2017 | 101360 | 323 | 319 | 2018 | 111496 | 444 | 398 |
| 33 | Makonde | 30.01 | -17.13 | 2015 | 156539 | 349 | 223 | 2016 | 159200 | 474 | 298 | 2017 | 161906 | 532 | 329 | 2018 | 178097 | 466 | 262 |
| 34 | Makoni | 32.18 | -18.36 | 2015 | 282407 | 80 | 28 | 2016 | 285513 | 328 | 115 | 2017 | 288654 | 292 | 101 | 2018 | 317519 | 608 | 191 |
| 35 | Mangwe | 28.01 | -20.98 | 2015 | 79850 | 289 | 362 | 2016 | 79850 | 325 | 407 | 2017 | 80249 | 273 | 340 | 2018 | 88274 | 216 | 245 |
| 36 | Marondera | 31.50 | -18.27 | 2015 | 187714 | 410 | 218 | 2016 | 190905 | 325 | 170 | 2017 | 194150 | 333 | 172 | 2018 | 213565 | 350 | 164 |
| 37 | Masvingo | 30.91 | -20.26 | 2015 | 311226 | 766 | 246 | 2016 | 314961 | 680 | 216 | 2017 | 318741 | 677 | 212 | 2018 | 350615 | 639 | 182 |
| 37 | Matobo | 28.49 | -20.96 | 2015 | 95408 | 227 | 238 | 2016 | 95885 | 190 | 198 | 2017 | 96364 | 241 | 250 | 2018 | 106000 | 235 | 222 |
| 39 | Mazowe | 30.93 | -17.26 | 2015 | 242087 | 541 | 223 | 2016 | 245234 | 574 | 234 | 2017 | 248422 | 443 | 178 | 2018 | 273264 | 437 | 160 |
| 40 | Mazowe | 30.93 | -17.26 | 2015 | 242087 | 541 | 223 | 2016 | 245234 | 574 | 234 | 2017 | 248422 | 443 | 178 | 2018 | 273264 | 437 | 160 |
| 41 | Mberengwa | 30.02 | -20.75 | 2015 | 191805 | 10 | 5 | 2016 | 193723 | 324 | 167 | 2017 | 195660 | 239 | 122 | 2018 | 215226 | 292 | 136 |
| 42 | Mbire | 30.52 | -16.12 | 2015 | 85145 | 88 | 103 | 2016 | 86252 | 68 | 79 | 2017 | 87373 | 31 | 35 | 2018 | 96110 | 101 | 105 |
| 43 | Mhondoro | 30.16 | -18.58 | 2015 | 104342 | 96 | 92 | 2016 | 104342 | 132 | 127 | 2017 | 114776 | 159 | 139 | 2018 | 126254 | 178 | 141 |
| 44 | Mount Darwin | 31.65 | -16.57 | 2015 | 220573 | 494 | 224 | 2016 | 223440 | 580 | 260 | 2017 | 226345 | 617 | 273 | 2018 | 248980 | 653 | 262 |
| 45 | Mudzi | 32.64 | -17.03 | 2015 | 139496 | 56 | 40 | 2016 | 141867 | 102 | 72 | 2017 | 144278 | 54 | 37 | 2018 | 158706 | 74 | 47 |
| 46 | Murewa | 31.82 | -17.79 | 2015 | 205204 | 329 | 160 | 2016 | 208692 | 341 | 163 | 2017 | 212240 | 310 | 146 | 2018 | 233464 | 381 | 163 |
| 47 | Mutare | 32.43 | -19.22 | 2015 | 194523 | 570 | 293 | 2016 | 196663 | 767 | 390 | 2017 | 198826 | 554 | 279 | 2018 | 218709 | 774 | 354 |
| 48 | Mutasa | 32.71 | -18.64 | 2015 | 175420 | 318 | 181 | 2016 | 177350 | 262 | 148 | 2017 | 179301 | 292 | 163 | 2018 | 197231 | 311 | 158 |
| 49 | Mutoko | 32.27 | -17.45 | 2015 | 153232 | 256 | 167 | 2016 | 155837 | 303 | 194 | 2017 | 158486 | 262 | 165 | 2018 | 174335 | 330 | 189 |
| 50 | Mwenezi | 30.69 | -21.37 | 2015 | 172270 | 1040 | 604 | 2016 | 174337 | 729 | 418 | 2017 | 176429 | 426 | 241 | 2018 | 194072 | 518 | 267 |
| 51 | Nkayi | 28.70 | -18.93 | 2015 | 111020 | 561 | 505 | 2016 | 111575 | 636 | 570 | 2017 | 112132 | 559 | 499 | 2018 | 123345 | 549 | 445 |
| 52 | Nyanga | 32.76 | -17.94 | 2015 | 129882 | 147 | 113 | 2016 | 131311 | 136 | 104 | 2017 | 132755 | 89 | 67 | 2018 | 146031 | 125 | 86 |
| 53 | Rushinga | 32.29 | -16.61 | 2015 | 73335 | 68 | 93 | 2016 | 74228 | 48 | 65 | 2017 | 75193 | 44 | 59 | 2018 | 82712 | 48 | 58 |
| 54 | Sanyati | 29.58 | -18.02 | 2015 | 112897 | 1024 | 907 | 2016 | 124187 | 736 | 593 | 2017 | 136605 | 787 | 576 | 2018 | 150266 | 1433 | 954 |
| 55 | Seke | 30.95 | -18.25 | 2015 | 106383 | 1075 | 1010 | 2016 | 108192 | 820 | 758 | 2017 | 110031 | 626 | 569 | 2018 | 121034 | 914 | 755 |
| 56 | Shamva | 31.64 | -17.10 | 2015 | 124253 | 103 | 83 | 2016 | 125868 | 212 | 168 | 2017 | 127504 | 271 | 213 | 2018 | 140254 | 200 | 143 |
| 57 | Shurugwi | 30.15 | -19.75 | 2015 | 102952 | 310 | 301 | 2016 | 103982 | 239 | 230 | 2017 | 105022 | 256 | 244 | 2018 | 115524 | 275 | 238 |
| 58 | Tsholotsho | 27.42 | -19.62 | 2015 | 115611 | 228 | 197 | 2016 | 116189 | 330 | 284 | 2017 | 116770 | 304 | 260 | 2018 | 128447 | 366 | 285 |
| 59 | Umguza | 28.28 | -19.82 | 2015 | 88839 | 237 | 267 | 2016 | 89238 | 209 | 234 | 2017 | 89684 | 207 | 231 | 2018 | 98652 | 252 | 255 |
| 60 | Umzingwane | 28.95 | -20.35 | 2015 | 63453 | 134 | 211 | 2016 | 63770 | 194 | 304 | 2017 | 64089 | 152 | 237 | 2018 | 70498 | 143 | 203 |
| 61 | UMP | 32.05 | -17.08 | 2015 | 117968 | 43 | 36 | 2016 | 119973 | 94 | 78 | 2017 | 122013 | 93 | 76 | 2018 | 134214 | 87 | 65 |
| 62 | Zaka | 31.43 | -20.39 | 2015 | 187704 | 279 | 149 | 2016 | 189956 | 358 | 188 | 2017 | 192235 | 295 | 153 | 2018 | 211459 | 219 | 104 |
| 63 | Zvimba | 30.45 | -17.51 | 2015 | 258222 | 726 | 281 | 2016 | 262612 | 437 | 166 | 2017 | 267076 | 629 | 236 | 2018 | 293784 | 557 | 190 |
| 64 | Zvishavane | 30.08 | -20.28 | 2015 | 118868 | 235 | 198 | 2016 | 120057 | 275 | 229 | 2017 | 121258 | 282 | 233 | 2018 | 133384 | 289 | 217 |
